# Remote effects of kidney drug transporter OAT1 on gut microbiome composition and urate homeostasis

**DOI:** 10.1172/jci.insight.172341

**Published:** 2023-11-08

**Authors:** Vladimir S. Ermakov, Jeffry C. Granados, Sanjay K. Nigam

**Affiliations:** 1 Department of Biology,; 2 Department of Bioengineering,; 3 Department of Pediatrics, and; 4 Department of Medicine, Division of Nephrology, University of California, San Diego (UCSD), La Jolla, California, USA.

**Keywords:** Nephrology, Chronic kidney disease, Epithelial transport of ions and water, Homeostasis

## Abstract

The organic anion transporter OAT1 (SLC22A6, originally identified as NKT) is a multispecific transporter responsible for the elimination by the kidney of small organic anions that derive from the gut microbiome. Many are uremic toxins associated with chronic kidney disease (CKD). OAT1 is among a group of “drug” transporters that act as hubs in a large homeostatic network regulating interorgan and interorganismal communication via small molecules. The Remote Sensing and Signaling Theory predicts that genetic deletion of such a key hub in the network results in compensatory interorganismal communication (e.g., host-gut microbe dynamics). Recent metabolomics data from *Oat1*-KO mice indicate that some of the most highly affected metabolites derive from bacterial tyrosine, tryptophan, purine, and fatty acid metabolism. Functional metagenomic analysis of fecal 16S amplicon and whole-genome sequencing revealed that loss of OAT1 was impressively associated with microbial pathways regulating production of urate, gut-derived *p*-cresol, tryptophan derivatives, and fatty acids. Certain changes, such as alterations in gut microbiome urate metabolism, appear compensatory. Thus, *Oat1* in the kidney appears to mediate remote interorganismal communication by regulating the gut microbiome composition and metabolic capability. Since OAT1 function in the proximal tubule is substantially affected in CKD, our results may shed light on the associated alterations in gut-microbiome dynamics.

## Introduction

The function of organic anion transporter 1 (OAT1/SLC22A6) is considered a rate-limiting step for the movement of many small negatively charged organic molecules from the blood into the urine (1). Originally discovered by our group as novel kidney transporter (NKT), OAT1 was one of the original SLC and ABC drug transporters highlighted by the FDA for testing of interactions with new drug entities (1–3). This is due its role in excreting many drugs (e.g., antibiotics, antivirals, NSAIDs, diuretics) (2, 4, 5). More recent studies have uncovered its critical role in regulating endogenous pathways involved in systemic and renal metabolism as well as signaling. In vitro transport data and in vivo KO of *Oat1* in mouse models have been used to identify the endogenous metabolites that are handled by this transporter. These studies (6–9) reveal that many of the metabolites altered in *Oat1*-KO mice originate from gut microbes.

OAT1*,* along with other SLC and ABC “drug” transporters (e.g., OATP1B1, MRP2, ABCG2), is considered to be a key systemic and organ hub in a proposed Remote Sensing and Signaling Network consisting of > 500 proteins (2, 10, 11). The Remote Sensing and Signaling Theory (RSST) posits that this network of genes — which partly overlaps with genes regulating drug absorption, distribution, metabolism, and excretion (ADME) — serves to maintain homeostatic balance of a broad range of endogenous small molecules in the body (2, 12, 13). An example of the Remote Sensing and Signaling System in action in human pathophysiology occurs when kidney function is diminished during chronic kidney disease (12). When the kidney is no longer able to effectively handle urate, a common end-product of purine metabolism and antioxidant, the accumulation of urate can lead to gout, hypertension, and progression of renal and cardiovascular disease (14). As a result, the intestine alters the expression and/or function of ABCG2 and partly remotely compensates for the damaged kidney, eliminating urate from the blood by excreting it into the gut lumen (15).

The Remote Sensing and Signaling network extends beyond communication along the gut-liver-kidney axis (16); recent work (17–19) supports its importance in other axes, such as the gut and adipose tissue, the gut and brain, the kidney and skin, the gut/kidney/heart axis, and between the host and the commensal microbes living within its gastrointestinal system. Previous work (20) has shown that changes in the gut microbiome composition influence the metabolic profile of the host organism. Furthermore, metabolites originating from microbes are substrates/inhibitors for proteins in the Remote Sensing and Signaling network and ADME network (transporters and enzymes), and many of these compounds can serve as ligands for nuclear receptors and GPCRs, which can impact gene expression in several tissues within the host (11, 21, 22).

Indeed, OAT1 in the kidney appears to be a major determinant of systemic levels of gut microbe–derived metabolites and signaling molecules (6–9). This relationship between host and gut microbiome is an important tenet in RSST, as reciprocal interorganismal communication has a considerable impact on host small-molecule homeostasis (11). The microbiota also serves as an initial interface between the dietary compounds and the host organism (23). The capacity of the microbiome to metabolize certain compounds over others greatly influences the metabolic profile of the host and plays a role in determining how the host organism, in turn, responds to the diet by the expression of various ADME and other proteins that are part of the Remote Sensing and Signaling System (10, 20, 24).

There is tremendous clinical and basic science interest in the role of gut microbes in kidney disease, among many other clinical syndromes (25–28). This interest is partly related to the wide range of small molecules produced by the gut microbiota that enter the circulation and that have been implicated as “uremic toxins” associated with negative outcomes in chronic kidney disease (CKD) (15, 29, 30). In the context of drug development, many of these gut-derived compounds have been suspected to interact with a variety of small-molecule drugs at the site of the transporter (31). Thus, it is not surprising that gut microbe–derived small molecules (some of which are uremic toxins) are transported by multispecific drug transporters in the gut, liver, kidney, and brain (7, 32–35). Indeed, in chronic kidney disease, there appears to be some type of cross-talk, possibly involving drug transporters and ligand-activated transcription factors, between the failing kidney and the gut microbes, resulting in alterations in the gut microbiota (36–38).

However, a particular multispecific drug transporter in the kidney has not been directly linked to alterations in the microbiota — and the attendant effects on systemic metabolism. OAT1 is perhaps the prototypical multispecific “drug” transporter in the kidney, handling a very broad range of endogenous and exogenous compounds that are organic anions (13). Genetic KO of *Oat1* in mice leads to the alteration of hundreds of metabolites in the blood, many of which are derived from the gut microbes and include uremic toxins widely considered to worsen the symptoms and progression of disease (7, 26, 32, 39, 40).

For example, tryptophan and tyrosine derivatives, such as indoxyl sulfate and *p*-cresol sulfate, are common gut-derived small molecules that require OAT1/OAT3 to be eliminated into the urine (2, 8). Accumulation of these compounds in the blood has been linked to renal fibrosis and the activation of proinflammatory cytokines, which leads to vascular injury and coronary artery disease (2, 21, 28, 41). Serum concentrations of both compounds serve as predictors for chronic kidney disease and heart disease progression, and a major research and pharmaceutical effort is aimed at developing therapeutic approaches to reduce the levels of these compounds (2, 21, 28, 41). Similarly, other gut microbe–derived uremic toxins transported by OAT1/OAT3 have also been found to be detrimental to remote tissue if not properly excreted from the body (21, 41).

Interestingly, while in vivo loss of *Oat1* in mice considerably increases serum concentrations of numerous small-molecule toxins thought to cause organ and vascular damage, these KO mice have a normal life span and present no clear evidence of organ dysfunction. That said, the double KO of rat *Oat1* and *Oat3* (*SLC22a8*) does result in impressive renal dysfunction and high levels of uremic toxins at 4 weeks of age, although this appears to mostly resolve by 7 weeks, apparently due to compensatory expression in the proximal tubule of another gut-derived uremic toxin transporter, *Oatp4c1* (*SLCO4c1*).

Transcriptomic analysis of the Oat1–single KO kidney has not revealed the significant upregulation of another transporter such as occurs in the double KO. This raises the question: Is there another compensatory mechanism that prevents the *Oat1*–single KO mice from becoming diseased despite the high levels of gut-derived small-molecule toxins?

RSST predicts a resetting of interorgan (e.g., kidney-liver) or interorganismal (e.g., host-gut microbes) communication would help restore homeostasis after genetic ablation of a key multispecific transporter necessary for the elimination of potentially harmful small molecules (2). Some compensatory mechanisms in the *Oat1*-KO organs have been revealed through genome scale metabolic reconstruction, but as mentioned, these do not clearly indicate a renal transport mechanism that would compensate for high levels of uremic toxins (9, 42, 43). Nevertheless, the gut microbiome’s immense potential to adapt in response to loss of *Oat1* in the single KO has not yet been examined. We thus sought to analyze how the loss of OAT1 in the kidney influences the composition and metabolic potential of the gut microbiota in relation to serum metabolites altered in the *Oat1* KO.

We discovered marked changes in the composition and metabolic capability of the gut microbiota that, together with recently published metabolomics data from the *Oat1* KO, provide a potentially new view of how OAT1 serves as a key interface between the gut microbes, the kidney, and systemic metabolism (8). Furthermore, our findings are relevant for understanding host-microbiome dynamics that may be altered in CKD since OAT1-mediated elimination of uremic toxins at the site of the proximal tubule is affected by the decline of renal function in CKD (38). Our results, connecting the loss of OAT1 to alterations in gut microbiome composition and function, may thus be helpful for understanding altered host-gut microbiome dynamics in CKD.

## Results

### 16S amplicon sequencing reveals Oat1-KO mice have greater microbiome alpha diversity than WT mice.

**Figure 1:**
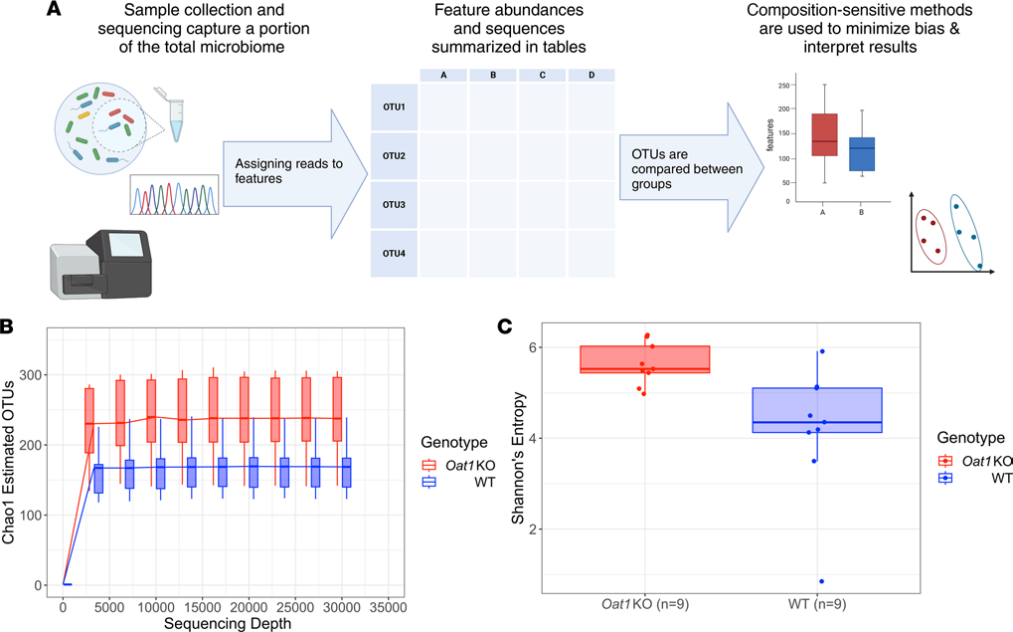
*Oat1-*KO and WT microbiomes have different alpha diversity metrics. 16S amplicon sequencing reveals differences in richness and evenness between the *Oat1-*KO (*n* = 9) and the WT mice (*n* = 9). (**A**) Summary of methods and workflow for generating 16S amplicon sequencing results. (**B**) Rarefaction curve comparing Chao1 indices at various depths. Chao1 estimated richness is largely unaffected at depths greater than 10,000 reads. Median Chao1 estimated OTUs were found to be 238 and 169 for the *Oat1* KO and WT, respectively. (**C**) Box plot of Shannon’s entropy of the 2 groups, with a median Shannon’s entropy of 5.52 for the *Oat1* KO and 4.35 for the WT.

We aimed to explore the differences in microbiome composition by analyzing the bacterial species present in fecal samples of mice that reflected the gut bacteria composition. Feces were collected from the *Oat1-*KO and WT mice, and bacterial DNA was extracted for 16S amplicon sequencing. Raw 16S reads were preprocessed and clustered into operational taxonomic units (OTUs) based on sequence similarity. An OTU represents a distinct member within the microbial community, where reads with greater than 97% similarity are considered to belong to the same OTU. We then applied denoising/error correction to improve the assignment of OTUs from reads (44). OTU assignment was performed using the *Qiime2* platform (45) and was summarized in a feature table (Supplemental Table 1; supplemental material available online with this article; https://doi.org/10.1172/jci.insight.172341DS1) (Figure 1A).

To assess the differences between the microbial communities, we first calculated the alpha diversity of the samples. Alpha diversity metrics are used to describe the total number of members in a community (richness) and how evenly they are distributed (evenness). Two commonly used metrics are the Chao1 index, which estimates the total members in a community based on the observed richness and composition of a sample, and Shannon’s entropy, which quantifies both richness and evenness.

A rarefaction curve using Chao1 indices calculated at various depths was generated (Figure 1B). Total richness is largely unaffected at depths greater than 10,000; thus, we do not anticipate our results to be affected by undersampling. Furthermore, it was found that the median Chao1 estimated OTUs in the *Oat1*-KO and WT controls were 238 and 169, respectively. Shannon’s entropy was also calculated as a measure of diversity (Figure 1C), and we found greater richness and evenness in the *Oat1-*KO compared with the WT mice (Kruskal-Wallis H test, *P* < 0.05; Table 1). Both median observed and estimated richness were greater in the *Oat1-*KO compared with the WT controls, with both metrics reaching statistical significance (Kruskal-Wallis H Test, *P* < 0.05; Table 1).

Enrichment of both Chao1 and Shannon’s entropy in the *Oat1* KO indicates an overall increase in alpha diversity in the KO compared with the WT controls, raising the possibility of important functional differences in the KO microbiome. To further explore what distinguished the 2 gut microbiomes, we investigated the beta diversity, which relates to differences within groups, to determine if there were any taxonomical differences in the *Oat1-*KO and WT microbiomes.

### Oat1-KO and WT mouse microbiomes have compositional differences.

**Figure 2:**
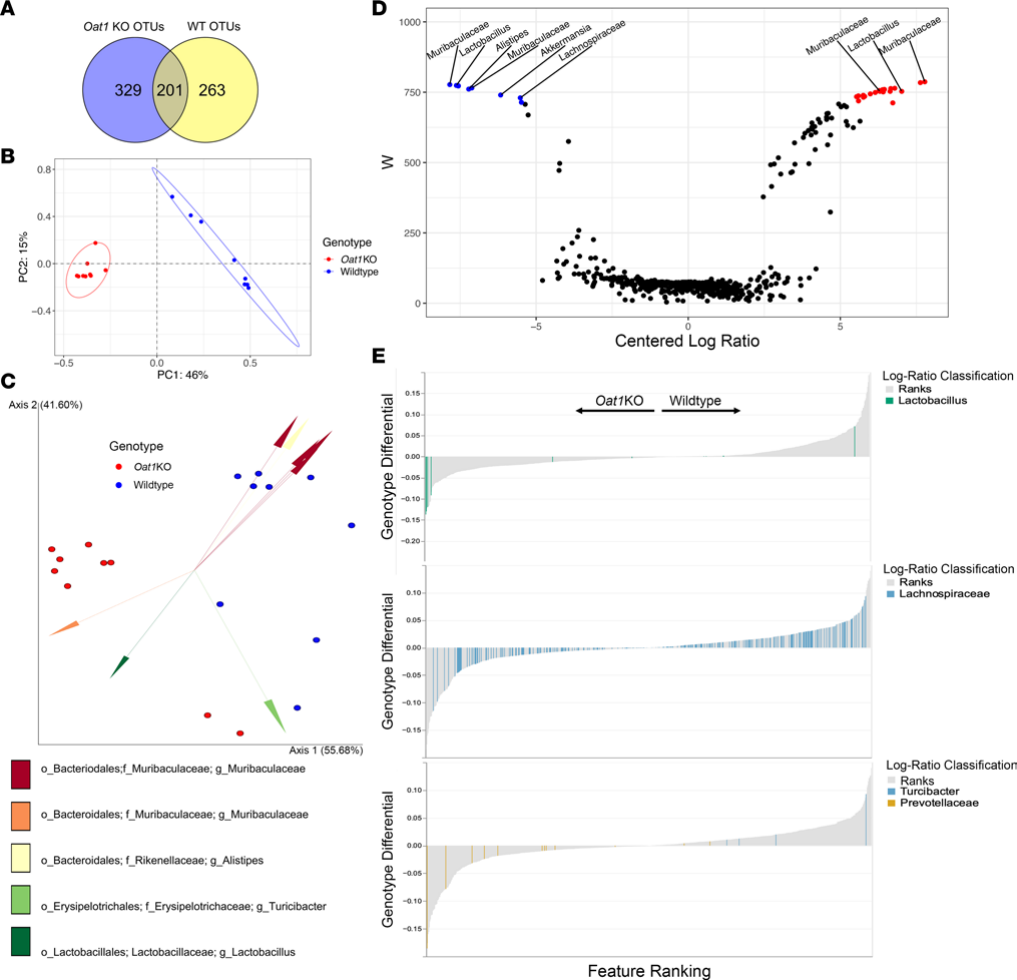
Beta diversity analysis reveals compositional differences between the *Oat1-*KO and WT gut microbiomes. (**A**) Venn diagram of OTUs assigned to *Oat1-*KO and WT samples (*n* = 9 each). (**B**) Principal coordinate analysis (PCoA) based on Bray-Curtis distances. The 95% CI ellipses are included for both groups. PC1 and PC2 account for 61% of variability in the species between the groups. (**C**) Biplot based on *Deicode*, a modified Aitchison distance calculation. Arrows represent the top 8 species that contribute to the variability in beta diversity. (**D**) Analysis of Compositions of Microbiomes (ANCOM) volcano plot of the *Oat1-*KO and WT OTUs. Features above a W statistic of 708 were deemed significant. Values colored blue represent features significantly increased in the WT, and those colored red represent features significantly elevated in the *Oat1* KO. Clr is the centered log-ratio of the KO with respect to WT. Thirty features were found to be significantly altered between the 2 groups, with many of the features belonging to the family *Muribaculaceae*. Members belonging to *Alistipes* and *Akkermansia* were elevated in the WT. Abundance of archaea was not measured. (**E**) Microbial differential ranks estimated from multinomial regression with *Prevotellacae, Turicibacter, Lactobacillus*, and *Lachnospiraceae* highlighted. The *y* axis represents the log-ratio of abundance between KO and WT samples, and the *x* axis numerically orders the ranks of each taxon in the analysis; ranks further down the *x* axis represent greater abundance in WT microbiomes, with respect to other taxa. Features belonging to *Turicibacter* appear to constitute a greater proportion of the WT microbiomes when compared with the *Oat1* KO, while *Prevotellacae* constitute a greater proportion of the *Oat1-*KO microbiomes. *Lactobacillus* appears to make up a greater proportion of the *Oat1-*KO microbiomes. *Lachnospiraceae* does not favor either genotype.

Beta diversity is used in microbiome studies to measure differences in taxonomic composition between bacterial communities. Due to previously reported changes (7, 8, 32) in circulating gut microbiome–derived compounds in the very same mice, we analyzed compositional differences in the *Oat1-*KO and WT microbiomes in the context of the serum metabolomics data; this could help determine whether the gut microbiome in the KO mice was reflecting changes in systemic metabolism due to loss of OAT1, which is almost exclusively expressed in the kidney. As a first step, we compared the OTUs present across the genotypes (Figure 2A) and generated a Bray-Curtis distance matrix in *Qiime2* to compare reads from *Oat1*-KO and WT samples (45, 46). The ordination of these results shows that *Oat1-*KO and WT samples form their own distinct clusters (Figure 2B). A biplot was then generated using *Deicode* (46, 47) robust Aitchison distances as a compositionally sensitive approach to measuring beta diversity (Figure 2C). These results indicate that *Oat1*-KO and WT vary in taxonomical composition of their communities, with members belonging to *Lactobacillus,* *Turicibacter*, *Muribaculaceae*, and *Prevotellaceae*, among others, explaining the differences in beta diversity in the 2 groups (Figure 2B).

To further investigate differences in taxa resulting from the deletion of *Oat1*, we performed an Analysis of Compositions of Microbiomes (ANCOM) as a composition-sensitive approach to determine differential abundance (Figure 2C) (48). The results of the ANCOM indicate 30 taxa to be significantly altered between KO and WT, many of which belonged to *Muribaculaceae*, a member of the phylum *Bacteriodetes* and are classified as contributors to the metabolism of complex polysaccharides in the gut (49). We analyzed the log ratios of the OTUs detected with respect to mouse genotype in the form of a rank plot (Figure 2D) (50). We found that *Turicibacter* ranked highly in the WT mice, indicating that it likely constitutes a greater proportion of the WT microbiome, compared with the *Oat1* KO (Figure 2D). *Prevotellaceae* and *Lactobacillus*, on the other hand, appeared to be dominant in *Oat1-*KO microbiomes. These differences in taxonomical composition led us to compare the metabolic potentials of the microbiomes, since these bacterial species are associated with distinct metabolic functions based on their genomes.

### Functional analyses of bacterial 16S and whole-genome sequencing results help explain OAT1-mediated changes in the serum metabolome.

We aimed to analyze the functional differences in the *Oat1-*KO microbiomes using 16S amplicon and whole-genome sequencing (WGS) to predict the metabolic consequences of the distinct microbiomes using metagenomic genome reconstruction methods. For functional relevance, we observed the bacterial changes in the context of serum metabolomics results from *Oat1-*KO mice across multiple experiments. These mice, while healthy and fertile, display dozens of significantly elevated circulating metabolites, suggesting that loss of OAT1 prevents their normal urinary clearance or otherwise plays a role in regulating their levels. In addition, we reanalyzed the metabolites consistently altered across several previous studies (Table 2). Among those that were consistently altered in multiple experiments, many belonged to tyrosine, fatty acid, tryptophan, and purine metabolism subpathways. We chose to focus on these specific subpathways in the analysis of altered genes/pathways in the microbiome.

**Figure 3:**
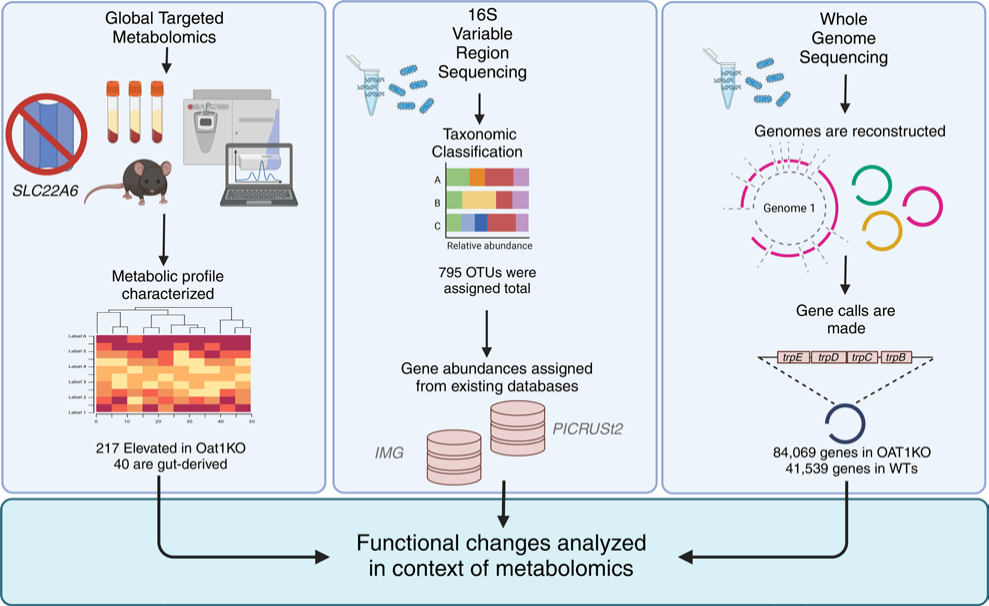
Schematic of functional analysis of *Oat1-*KO and WT microbiomes. Serum from *Oat1-*KO and WT mice (*n* = 4 each) was collected and used for global targeted metabolomics analyses. In total, 217 metabolites were found to be significantly elevated in the KO, 40 of which were characterized as gut microbiome derived. 16S amplicon sequencing was performed on feces collected from the mice (*n* = 9 *Oat1* KO, *n* = 9 WT). In total, 795 OTUs were reconstructed across both groups. Functional profiling was accomplished using PICRUSt2, which utilizes the Integrated Microbial Genomes (IMG) database to assign genes to OTUs. WGS reconstructed 112 genomes in the *Oat1* KO and 59 genomes in the WT (*n* = 7 *Oat1* KO, *n* = 8 WT). The genomes reconstructed in the *Oat1-*KO microbiomes were annotated with 84,069 KEGG gene IDs, and the WT mice were annotated with 41,539 KEGG genes. Abundance of genes in the microbiomes was used as a measure of functional enrichment and was interpreted in the context of serum metabolomics.

Previous work (8, 9, 21) has also suggested that OAT1 plays a crucial role in mediating the communication between the host and the gut microbes, via regulation of gut-derived metabolites. This suggests that the gut microbiome may adjust its composition and function in response to loss of OAT1; thus, we aimed to study the bacterial species present and their metabolic capacities using both 16S sequencing and WGS of the microbiota. 16S amplicon sequencing seeks to classify members of a bacterial community based on their 16S variable regions. While this method does not directly measure functional enrichment, the recently classified taxa can be annotated with genes based on established WGS databases, such as the Integrated Microbial Genomes (IMG) database (Figure 3). However, this method may introduce bias in functional predictions by covering only species that have been previously sequenced. WGS remedies this issue by directly sequencing the entirety of the genomes in a community. Functional enrichment can then be predicted by measuring the abundance of genes across the genomes recovered. For our analysis of the *Oat1-*KO and WT control microbiomes, we decided to investigate functional changes based on our 16S amplicon sequencing results and then perform WGS and metagenomic genome reconstruction to achieve better resolution on the genes altered. Given that many of the gut microbe–derived products altered in the *Oat1-*KO serum, including indoxyl sulfate and *p*-cresol sulfate, have production that is rate limited by only a handful of enzymes in the bacteria, the resolution offered by WGS proved useful for interpreting functional changes in the microbiome in the context of serum metabolites.

### Tyrosine metabolism in the microbiome in the context of serum metabolomics.

**Figure 4:**
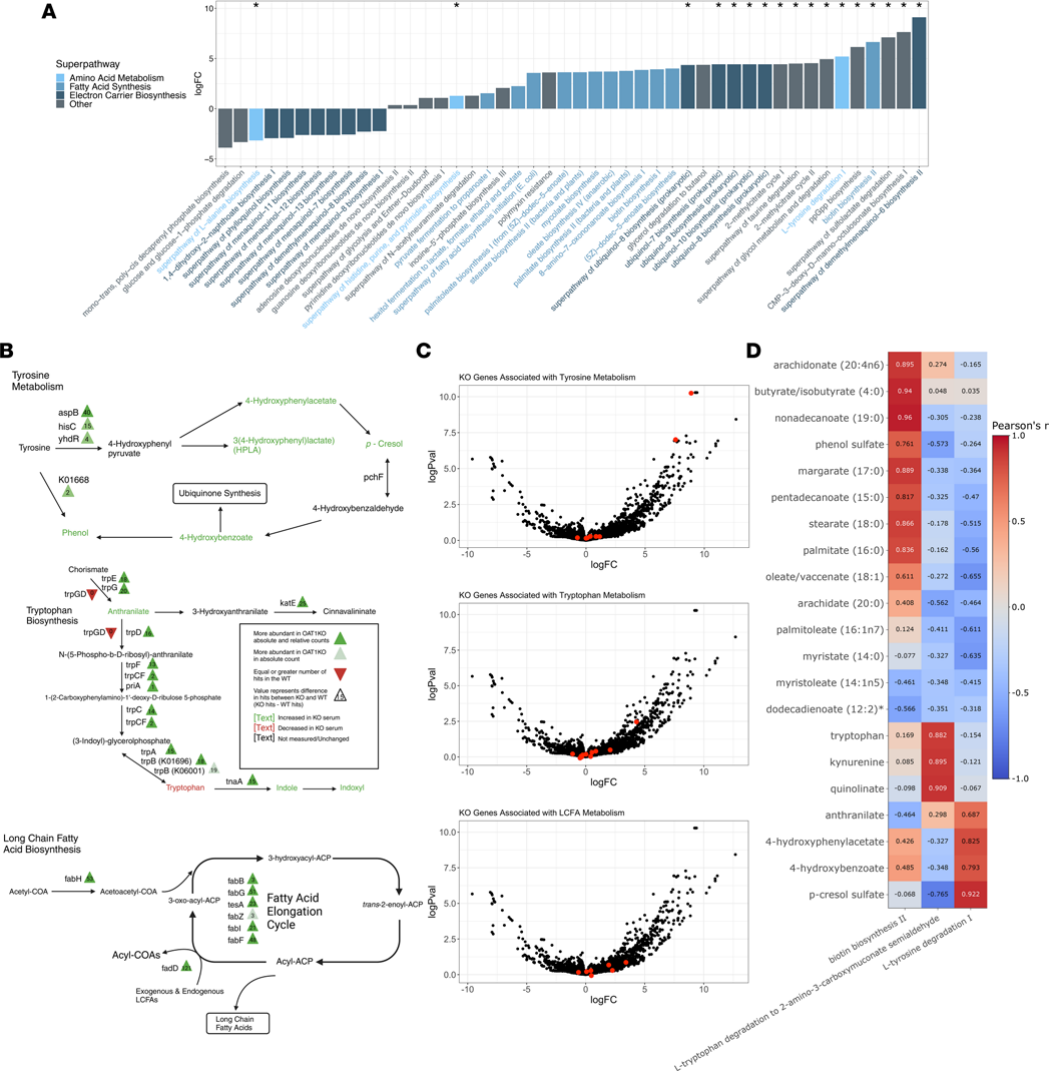
16S and WGS pathway analyses reveal microbial pathways enriched in the *Oat1* KO. (**A**) Bar chart of the predicted MetaCyc pathways from the 16S PICRUSt2 analysis of *Oat1-*KO and WT mice (*n* = 9 each). Pathways enriched in the *Oat1* KO are represented by a positive log fold-change (LogFC). A voom differential expression analysis was performed to generate fold-changes and *P* values. *P* values were adjusted for multiple comparisons using the Benjamini-Hochberg correction. Of 346 total detected MetaCyc pathways, those charted had an *P*_adj_ < 0.1. Pathways annotated with an asterisk represent those with an *P*_adj_ < 0.05. (**B**) Tyrosine degradation, tryptophan metabolism, and LCFA synthesis pathways summarizing the abundance of enzymes reconstructed from WGS metagenome. Enzymes with a > 2-fold increase in abundance in the KO were annotated with a dark green arrow. Integer values inside the arrows represent the difference in hits an enzyme had in the KO versus WT. Tyrosine degradation, tryptophan metabolism, and LCFA synthesis are favored in the *Oat1-*KO microbiomes. (**C**) Volcano plot of gene enrichment from KEGG ontology annotated genes in PICRUSt2. Red points highlight genes present in the adjacent pathway diagram (**B**). Positive log fold-changes reflect an increased abundance of a gene in the *Oat1* KO. Both WGS and 16S pathway analyses reflect increases in tyrosine degradation, tryptophan metabolism, and LCFA production in the *Oat1* KO. Fold-change and *P* values were generated using voom. (**D**) Serum abundance of metabolites from *Oat1-*KO (*n* = 4) mice were correlated with MetaCyc pathways detected from the 16S PICRUSt2 analysis. Cells in heatmap are annotated with a Pearson’s *R* correlation coefficient. Serum microbe-derived metabolites correlate well with associated pathway enrichment in the microbiome. Therefore, pathways in the microbiome may have a predictive value for serum concentrations of gut-derived compounds.

Functional analysis of 16S and WGS data were consistent with elevated tyrosine metabolism in the *Oat1-*KO microbiomes. 16S amplicon data were analyzed using PICRUSt2 (Phylogenetic Investigation of Communities by Reconstruction of Unobserved States, second iteration) (51) and were used to predict functional pathway enrichment in the microbiomes (Figure 3). Individual gene enrichments were assigned Kyoto Encyclopedia of Genes and Genomes (KEGG) IDs, and pathways were annotated using MetaCyc. Eighteen total pathways were found to be significantly altered between the *Oat1-*KO and WT mice, including some that were consistent with known OAT1 functional interactions in vitro and/or in vivo (8, 13, 15, 27). For example, tyrosine degradation I was significantly elevated in the KO (log fold-change [LogFC] > 5, adjusted *P* value [*P*_adj_] < 0.05; Figure 4A), and this describes the conversion of tyrosine to 4-hydroxyphenyl pyruvate. At the gene level, we found a significant elevation of tyrosine phenol-lyase, which catalyzes the conversion of tyrosine to phenol, precursor of a uremic toxin (LogFC > 9, *P*_adj_ < 0.05; Figure 4C). Interestingly, 4-cresol dehydrogenase, *pchF*, an enzyme responsible for the conversion of the uremic toxin precursor *p*-cresol to 4-hydroxybenzaldehyde, was also significantly elevated in the KO. Our serum metabolomics results show greatly elevated levels of *p*-cresol sulfate, so it seems plausible that the microbiome is responding to these altered serum metabolites by shifting its own metabolism to favor the degradation of the bacterial precursor to *p*-cresol sulfate (Figure 4B).

We then applied WGS to perform a deeper analysis of metabolic differences between the KO and WT gut bacteria. To accomplish this, we performed a metagenomic genome reconstruction, the process by which metagenomic contigs are assembled to re-create individual bacterial genomes. The genomes recovered from the greater library of contigs are referred to as metagenome-assembled genomes (MAGs) and represent the genomic makeup of 1 member of the microbiome. In total, we recovered 112 bins for the *Oat1* KO and 59 bins for the WT, with each bin representing a reconstructed genome or MAG (Figure 3). These MAGs were then functionally annotated using KEGG IDs. When summed, the number of hits a gene has reflects the abundance of this gene in the metagenome. Thus, a greater abundance of a particular gene is reflective of a propensity for the microbiome to engage in this metabolic task. In the case of the *Oat1* KO and WT, we considered a gene as enriched in the KO if there was an associated > 2-fold change in the number of hits.

Enzymes associated with tyrosine metabolism were reconstructed in greater numbers in the *Oat1* KO compared with the WT. Aspartate aminotransferase *aspB*, which can catalyze the conversion of L-tyrosine to 4-hydroxyphenyl pyruvate, was reconstructed 48 times in the KO genomes compared with the 8 reconstructed in the WT (Figure 4B). Downstream products of tyrosine metabolism, including 4-hydroxyphenylacetate and *p*-cresol sulfate, were elevated in the serum of the KO mice and could be resulting, at least in part, due to this greater tyrosine metabolism in the gut. Similarly, tyrosine phenol-lyase*,* which was also noted in the 16S analysis, was reconstructed in greater numbers in the KO compared with the WT, with 4 and 2 reconstructions of these genes, respectively (Figure 4B). As mentioned previously, this enzyme catalyzes the production of phenol from tyrosine, which can be later conjugated with sulfate during phase II metabolism to generate phenol sulfate, a potentially toxic metabolite (also a uremic toxin) elevated in serum of the KO mice, in part presumably due to changes in microbial metabolism.

To determine whether pathway enrichments from our 16S results correlate with serum concentrations of various gut-derived products, we performed a Pearson’s correlation using the metabolomics results from our *Oat1-*KO mice. We observed that the tyrosine derivative *p*-cresol sulfate correlates well with tyrosine degradation in the bacteria (*r* > 0.9; Figure 4D), while other tyrosine derivatives, such as 4-hydroxyphenylacetate and 4-hydroxybenzoate, also show a strong positive correlation with tyrosine metabolism, with *r* = 0.82 and *r* = 0.79, respectively. These results strongly suggest that the serum concentrations of these metabolites can be at least partially explained by altered gut metabolism. In the *Oat1-*KO mouse, tyrosine derivatives (e.g., *p*-cresol sulfate) are elevated in the serum, and it appears that the gut microbiome shifts its own metabolism to favor tyrosine degradation (i.e., increased *p*-cresol and phenols).

### Gut microbiome tryptophan metabolism appears connected to OAT1 regulation of tryptophan derivatives in the serum.

In addition to tyrosine derivatives, tryptophan metabolites have also been shown (40) to be handled by OAT1 in vivo and in vitro (Table 2). Many of these compounds — such as indoxyl sulfate, indolepropionate, and indolelactate — originate in the gut microbiome from the conversion of tryptophan to indole by tryptophanase-expressing bacteria. Many are also considered uremic toxins, and they also have an increasingly appreciated role in signaling (15, 27, 36). Hence, we sought to determine whether there was an existing relationship between tryptophan metabolism and the metabolic capacity of the gut microbiome.

Functional predictions from the 16S PICRUSt2 analysis did not show significant changes in tryptophan degradation or synthesis pathways. However, at the gene level, an enzyme involved in an intermediate step in tryptophan synthesis, indole-3-gylcerol phosphate synthase *trpCF*, was found to be significantly elevated in the KO (*P*_adj_ < 0.05, LogFC > 4; Figure 4C). Whole-genome reconstructions show a greater abundance of several enzymes responsible for the synthesis and degradation of tryptophan in the *Oat1-*KO. Bacterial tryptophanase catalyzes the production of the signaling precursor, indole, from tryptophan and was reconstructed in greater numbers in the KO, with 19 hits compared with 4 (Figure 4B). Tryptophan synthase A*,* which can lead to generation of indole, was reconstructed in 22 genomes in the KO compared with 4 in the WT (Figure 4B). The generation of indole by both pathways is likely associated with the downstream production of compounds like indoxyl sulfate, indolepropionate, and indolelactate, which are the products of liver metabolism of indole. These compounds have been shown to be affected by in vivo loss of OAT1 in mouse models, which were also supported by in vitro assays, suggesting a strong mechanistic relationship (32, 39). The increased production of indole-containing compounds is similar to what was observed for tyrosine derivatives, with the increased levels in the serum, likely owing to both increased microbial production and diminished clearance.

The abundance of bacterial pathways in *Oat1-*KO mice was correlated with the serum abundances of related metabolites to see how well bacterial tryptophan metabolism explains certain serum tryptophan-derived metabolites. We correlated one of the detected tryptophan degradation pathways, L-tryptophan degradation to 2-amino-3-carboxymuconate semialdehyde, with its closely associated metabolites. We found that tryptophan, kynurenine, and quinolinate all correlated well with this pathway (*r* > 0.75 each; Figure 4D). Kynurenine has previously been shown (39) to interact with OAT1 and becomes elevated in the serum of mice when *Oat1* is deleted. Quinolinate was particularly notable, since it is nonenzymatically generated from 2-amino-3-carboxymuconate semialdehyde, plays important roles in physiology, and is considered neurotoxic. Interestingly, “synthesis of quinolinate from tryptophan” was one of the few “metabolic tasks” found to be clearly altered in the kidney of the *Oat1*-KO mouse (39). While these metabolites are also produced by host metabolism, their correlation with this bacterial pathway indicates a potential combined effect between the host and microbiome in establishing serum levels of these metabolites involved in signaling. The increase in the bacterial tryptophan degradation pathway raises the possibility that the microbiome is also responding to an accumulation of potentially toxic tryptophan derivatives due to loss of renal clearance via OAT1.

Taken together, the data suggest a resetting in microbial tryptophan metabolism in response to loss of OAT1. Given the tryptophan synthesis and indole-producing degradation bacterial pathways reconstructed from WGS, this resetting is likely to apply to many of the large number of tryptophan derivatives other than indoxyl sulfate altered in the *Oat1-*KO serum. In light of a previous study (39) focused on altered systemic tryptophan metabolism and kidney tryptophan metabolism (by metabolic task analysis) in the *Oat1*-KO mouse, the results suggest a strong connection, via tryptophan metabolism, between the gut microbiome and the kidney through the function of the OAT1 transporter.

### Long-chain fatty acid–related pathways in the gut microbiome are affected by loss of OAT1.

Several long-chain fatty acids are elevated in the serum resulting from loss of OAT1 (Table 2); hence, we analyzed pathways associated with fatty acid metabolism. The 16S functional predictions indicate elevations of several long-chain fatty acid synthesis pathways, including palmitate, stearate, oleate, and palmitoleate biosynthesis; however, these only approached significance (0.05 < *P*_adj_ < 0.1), so they did not fully meet criteria. We then analyzed pathways critical to fatty acid synthesis. The most relevant of these was Biotin Biosynthesis 2, which was significantly elevated in the KO based on 16S results (Figure 4A, LogFC > 6, *P*_adj_ < 0.05). Biotin is necessary for fatty acid synthesis in bacteria and reflective of fatty acid synthesis in the microbiome, which could explain the elevated levels in the serum.

When the *Oat1-*KO microbiomes were reconstructed during WGS, we found many enzymes responsible for the initiation of fatty acid synthesis and fatty acid elongation to be reconstructed in greater numbers in the KO compared with the WT controls. For example, bacterial long-chain acyl-CoA synthase *fadD,* which converts endogenously produced and exogenous free fatty acids to acyl-CoAs, was reconstructed 146 times in the KO and 25 times in the WT. Long-chain acyl-CoAs were not measured in the metabolomics platform used; however, it is possible that serum acyl-CoAs may be affected by this change in the gut. Bacterial enzymes responsible for the elongation of fatty acids, such as 3-oxoacyl-ACP-synthase 2 *fabF,* were also more abundant in the KO, with 69 reconstructions in the KO and 21 reconstructions in the WT, suggesting increased long-chain fatty acid production in the KO (Figure 4B).

We then correlated serum levels of long-chain fatty acids with Biotin biosynthesis 2 as a general pathway for long-chain fatty acid synthesis, and we saw strong correlation of several serum long-chain fatty acids (*r* > 0.8; Figure 4D). More specifically, we saw serum arachidonic acid and pentadecanoic acid, which have been previously shown (40) to change in response to *Oat1* deletion in mice, correlated with Biotin biosynthesis 2 with a coefficient of 0.89 and 0.81, respectively. We also saw serum concentrations of stearate and palmitate also correlated well (stearate *r* = 0.866, palmitate *r* = 0.83) with biotin biosynthesis. While many of these long-chain fatty acids are elevated in vivo in the *Oat1* KO, the available IC_50_ values in vitro suggest a moderate level of interaction (40). Thus, it seems likely that the serum level changes of many of these long-chain fatty acids, some of which are involved in signaling, are in large part due to changes in metabolism of the gut bacteria.

### Urate production by the gut microbes is diminished following loss of Oat1.

Urate is a well-established in vivo substrate of OAT1 (14, 32, 52). Human polymorphisms in *OAT1* are associated with hyperuricemia (53, 54). OAT1 and OAT3 are considered the main route of entry of urate across the basolateral (blood-facing) side of the proximal tubule cell in the kidney (13). In patients with hyperuricemia and/or gout, it is now believed that the gut microbiome alters its production of urate in response to elevated systemic urate levels (54). RSST predicts that, as with ABCG2-mediated transport favoring excretion across the gut epithelia (12), there should be a change in the microbiome to maintain urate homeostasis since high levels are associated with toxicity and disease (54).

**Figure 5:**
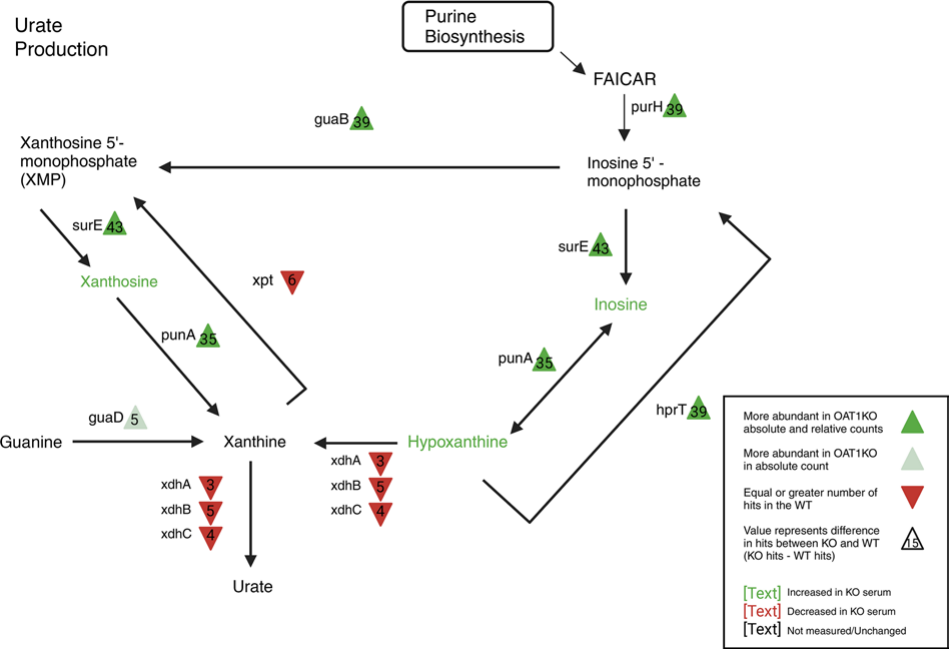
Pathway analysis of bacterial urate production. WGS reconstructs many of the enzymes responsible for purine and urate production in gut bacteria. Urate producing xanthine dehydrogenase was reconstructed in 5 bacterial genomes of the WT microbiomes and was not reconstructed in the *Oat1* KO, suggesting decreased urate production in the KO.

In the *Oat1* KO, we observed elevated serum levels of purines, especially inosine (8-fold) and hypoxanthine (16-fold) (Table 2). Although we did not analyze urine in this study, in our previous studies (32, 52) of the *Oat1* KO, renal urate secretion was diminished and urine urate levels were low, consistent with systemic retention. We thus analyzed urate synthesis and downstream pathways in the context of broader purine metabolism in gut microbiome of the *Oat1* KO. As shown in Figure 5, most of the bacterial enzymes involved in purine metabolism were altered in the *Oat1* KO. Notably, we observed a decrease in urate production (5 genomes in the WT compared with 0 in the *Oat1* KO) (Figure 5). Moreover, analysis of the composition of the gut microbiota showed an increase in *Prevotellaceae* (Figure 2E), which is also seen in patients with hyperuricemia/gout (55). This suggests that gut microbiome remodeling could be occurring to compensate for elevated purines and/or urate in the *Oat1* KO.

## Discussion

**Figure 6:**
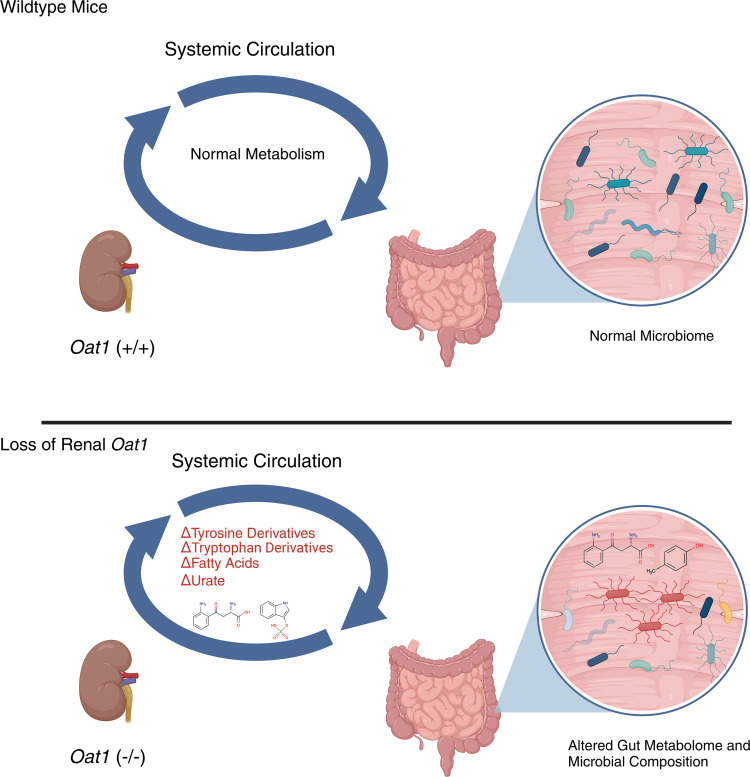
Illustration of how loss of *Oat1* may alter gut microbiome composition. Altered renal clearance of gut-derived compounds and organic anions through loss of *Oat1* leads to changes in serum metabolites. Systemic changes in organic anions may change the composition and function of the commensal microbiome. Some of the functional differences observed, due to loss of OAT1, include changes in tyrosine degradation, tryptophan degradation, long-chain fatty acid synthesis, and urate production.

While the effect of gut bacteria on the host has been of great interest for some time, there has been little research on how host metabolites transported by multispecific “drug” transporters influence the composition of the microbiome. Also largely unexplored is the role of these transporters in mediating crosstalk between the microbiome and a particular organ like the kidney, which handles the elimination of many gut microbe–derived metabolites via OAT1. Here, we have analyzed bacterial metagenomics in the context of metabolomics in mice in which a major drug transporter has been genetically disrupted. Furthermore, the fact that OAT1 is almost exclusively located in the kidney proximal tubule makes it possible to consider the relationship of an important aspect of physiology in a single organ to gut microbiome dynamics. Our results support the notion of remote communication between the kidney and the bacterial species within the gut via OAT1. The changes in the gut microbiome composition and metabolic capacity associated with the loss of renal OAT1 function help explain resulting changes in serum metabolomics (Figure 6), particularly in the context of systemic urate, tyrosine, tryptophan, and long-chain fatty acid metabolism.

Principal ordination shows distinct clustering of beta diversities in the *Oat1-*KO and WT mice, indicating that genetic differences in the mice can help explain compositional shifts in the gut microbiome. We found 30 OTUs to be significantly altered between the KO and WT controls, with compositional shifts favoring members of *Prevotellaceae* and *Lactobacillus* in the *Oat1* KO. Interestingly, previous work (56) has shown that both *Prevotellaceae* and *Lactobacillus* are responsible for saturated long-chain fatty acid production by commensal microbes, a result also supported by our functional analysis of the 16S amplicon and WGS data. We found enrichment in long-chain fatty acid production in the *Oat1* KO in terms of PICRUSt2 pathway enrichment as well as a greater number of fatty acid–producing *fab* genes reconstructed in the KO (Figure 4B). Moreover, tryptophan and tyrosine degradation were also found to be enriched in the KO microbiomes, again with greater reconstructions of tryptophan- and tyrosine-degrading enzymes.

Taken together, the findings are of particular interest when analyzed in the context of several of our previous studies (32, 39, 40) of the serum metabolites altered in the *Oat1-*KO mice, which have elevations of several LCFAs, tryptophan, tyrosine derivatives, and urate (Table 2). It is important to reemphasize here that nearly all of the serum metabolomics data referred to in the text are from the same mice on which the fecal metagenomics performed.

Particularly interesting is the apparent collaboration between gut microbes and OAT1 in the context of uric acid homeostasis. Enzymes associated with bacterial urate production were not detected in the KO but were found in the WT, suggesting diminished urate production as compensation for the lack of OAT1. The effect the microbiome has on urate levels has to be considered in light of the ability of mice to convert urate into the more water-soluble allantoin — a pathway not available to humans. Nevertheless, it is interesting to note that changes to the microbiota have also been reported (6, 55) in patients with hyperuricemia; this is often associated with renal underexcretion of urate, which also occurs in the *Oat1-*KO mouse. Our results suggest that the microbiome may be reducing its urate production in an attempt to restore urate homeostasis, as laid out in RSST (12, 14, 15).

While the aforementioned results indicate that the various other bacterial metabolic pathways (involving tyrosine, tryptophan, and long-chain fatty acids) are altered in the *Oat1-*KO mice, the potential compensatory value for the murine host is sometimes not as obvious as in the case of urate. To explore the relationship between serum levels of gut-derived products and bacterial pathway enrichment, we analyzed pathway abundances in the *Oat1*-KO microbiomes with serum metabolites and found that tyrosine, tryptophan, and long-chain fatty acid–producing pathways correlated well with their associated products. Together with evidence of increased degradation of tryptophan (i.e., increased indole) and tyrosine (i.e., increased *p*-cresol and phenol), this supports the view that the microbiome is at least in part responsible for, and/or is responding to, the altered serum metabolites relating to tyrosine, tryptophan, and long-chain fatty acid metabolism in the host.

In the case of tryptophan and tyrosine derivatives, which serve as organic anion substrates for OAT1 and, thus, are in part likely elevated largely due to diminished renal clearance, the gut microbiome may be shifting its metabolism of tryptophan and tyrosine and their many derivatives. Some of these derivatives may be beneficial — and involved in key signaling events between the microbiome and various organs — while some of these may be harmful, and others may have a dual beneficial-harmful nature related to their levels in the tissue in question (27). In the case of LCFAs, it appears that the microbiome plays a larger role in establishing serum concentrations of these metabolites than loss of renal elimination via OAT1. Whether the microbiome’s production of LCFAs in the *Oat1* KO serves a protective compensatory role in various tissues remains to be determined.

The end result of the gut microbiome changes in response to the loss of OAT1 seems to be a “resetting” of small-molecule interorganismal (e.g., host-gut microbes) communication that, as seems most clear in the case of urate, helps restore homeostasis after loss of function of a “drug” transporter, OAT1 (SLC22A6). OAT1 is a key multispecific organic anion transporter in a gene family (SLC22) functioning as a hub in a Remote Sensing and Signaling Network of proteins regulating the aforementioned small molecules and many others (10). We hypothesize that this is a general paradigm for many other multispecific “drug” transporters of both the SLC (e.g., OATs, OCTs, OATPs) and ABC (e.g., BCRP or ABCG2, MRP or ABCC, Pgp or ABCB1) families. Due to their multispecificity, a dozen or so such “drug” transporters, together with drug metabolizing enzymes (Phase I and Phase II), have the potential to handle the bulk of small organic anions generated by the gut microbiome.

This hypothesis implies that multispecific “drug” transporters other than OAT1 deserve intense further investigation because, if results similar to what we find with OAT1 are obtained, there is the potential to substantially revise our views on mechanisms within the body underlying the regulation of microbiome-host metabolism. It also has clinically actionable consequences for understanding drug-metabolite interactions and the disposition of endogenous metabolites of pathophysiological significance such as gut microbe–derived uremic toxins and, likewise, those accumulating in hepatic disease and metabolic syndrome. Indeed, many of the gut microbe–derived metabolites described here are also uremic toxins thought to be responsible for the progression of CKD and CKD-associated cardiovascular disease (15, 25, 27, 28).

In rats with a CRISPR/Cas9-mediated double KO of OAT1 (Slc22a6) and OAT3 (Slc22a8), renal dysfunction was observed in 4-week-old rats, but this was ameliorated by 7 weeks (57). This was apparently due to a compensatory increase in renal SLCO4C1 expression by 7 weeks (57). Previous work has shown that overexpression of the OATP transporter, SLCO4C1, has a protective effect on 5/6 nephrectomy rats modeling CKD by reducing systemic uremic toxin levels (58). Diminished tubular secretion in the kidney is characteristic of CKD (25, 27). Like SLCO4C1, OAT1 serves as an important uptake transporter at the site of the proximal tubule, and its genetic deletion in mouse models leads to decreased tubular secretion and its associated changes to metabolism (8, 15). Given that many of the gut-derived metabolite changes in our *Oat1-*KO mice are reflected in the serum of patients with CKD, our findings may be highly relevant to understanding changes in host-microbiome dynamics that arise in the context of declining renal function. The importance of OAT1 in handling uremic toxins in CKD is clear (7, 8, 38), but renal function appears preserved, presumably because OAT3 expression and the gut microbiome compensates. Although a significant increase in SLCO4C1 has not been reported in the *Oat1* KO, this may not come into play unless both OAT1 and OAT3 are not expressed, as was the case in the rat double KO. The implication is that upregulating OAT expression, perhaps together with manipulation of the microbiome, could help slow the progression of CKD (15).

In summary, genetic deletion of *Oat1* leads to a number of definable changes in the bacterial composition of the gut microbiome. OAT1 in the kidney plays a key role in interorganismal small-molecule communication between the host and gut microbiome, particularly involving metabolites handled by these altered bacterial species. Our data and analysis indicate that the gut microbiome dynamics, together with OAT1 transport function, help maintain systemic homeostasis by regulating host pathways involving urate, tryptophan and tyrosine derivatives, long-chain fatty acids, and other metabolites (8).

## Methods

### Animals.

Nine adult *Oat1*-KO and 9 WT C57BL/6 mice were housed in cages under a 12-hour day-night cycle with ad libitum access to food and water. *Oat1*-KO mice were generated and maintained as previously described (59). For 4 weeks, feces were collected weekly from each mouse in a sterile field and immediately snap frozen. Feces samples were kept at –80°C until further analysis. Whole blood was collected via terminal cardiac puncture, and serum was extracted, snap frozen, and stored at –80°C for metabolomics analysis as previously described (8).

### Metagenomic analysis.

Sequencing, library preparation, and isolation of genetic material were all performed by the UCSD Microbiome Core. Extraction protocols match those established in the Earth Microbiome Project (60). Whole genome and 16S libraries were prepped and sequenced on an Illumina platform. Reads were analyzed using the sequencing study manager *Qiime2* (45). A default workflow for 16S amplicon sequencing results was run. Trimming, demultiplexing, and deblurring of reads were all handled by *Qiime2*. A feature table containing OTU abundances and their reference sequences was generated and used for subsequent analyses. Alpha and beta diversity metrics were calculated in the *Qiime2* platform. A minimum depth of 45,632 was found across all samples. This depth was selected for rarefaction when necessary. The ANCOM was run in *Qiime2* (45, 48). Denoised features were assigned taxonomies using the Silva r138.1 rRNA database (61). The distance matrix used for ordination was generated using an *Deicode*’s Robust Aitchison distance calculation due to its sensitivity to compositional data (47, 48). Ordinations and alpha diversity graphs were plotted using ggplot2 in the R environment. *Qurro* (50) was used for the multinomial differential analysis and to generate the rank plot.

### Functional abundance predictions.

16S rRNA sequences for OTUs were input into PICRUSt2 (51), a bioinformatics tool that uses reference genomes to predict functional abundance. Outputs contained tables with abundance values of MetaCyc pathway and KEGG ontology genes for each sample.

### WGS metagenomics.

In total, 72 million reads were sequenced for the WT and 40 million reads for the KO. Raw paired-end reads were processed using Trimmomatic (v0.39) (62). FastQC was run on the trimmed reads, assessing GC content, per-base quality, and read duplication. Eight of 9 *Oat1-*KO and WT mice were selected for fecal WGS. One pair of *Oat1-*KO reads was found to be unsatisfactory due to having high duplication rates and large GC content spikes. This library was removed from the WGS analysis, leaving 7 *Oat1-*KO samples and 8 WT samples. Seventy-nine million reads were retained in the *Oat1* KO, and 56 million were retained in the WT. WT and *Oat1-*KO metagenomes were coassembled using MegaHIT (v1.2.9) (63), with a minimum contig length of 1 kb, with 77,116 contigs constructed in the KO and 48,295 contigs created in the WT — a total length of 290 million and 157 million bp, respectively. Contigs were then binned using metabat2 (v2.15) (64), generating 112 bins for the *Oat1* KO and 59 bins for the WT. All bins were retained in the analysis. Open reading frames were predicted using Prodigal (v2.63) (65). Assembled genomes were annotated with KEGG IDs using KofamKOALA (66). The number of hits across all bins was counted and compared between the 2 groups.

### Metabolomics of KO versus WT mice.

The relevant metabolomics methods and analyses have been previously described (8), and here we have focused on a subset of all significantly altered pathways that are necessary for understanding the microbiome results. Briefly, samples of serum from KO and WT mice were delivered to Metabolon. According to the metabolomics protocol provided by Metabolon, serum proteins were removed and samples were subjected to ultra–high-performance liquid chromatography–tandem mass spectroscopy (UPLC-MS/MS). after passing quality checks. This is a targeted protocol. Peaks were identified based on retention time, mass/charge ratio, and known spectra; they were quantified by AUC.

### Statistics.

Statistical significance was determined for alpha diversity metrics using the Kruskal-Wallis H test, with *P* < 0.05 being considered significant. In the 16S functional analysis, when making comparisons between genes or between pathway abundances, fold changes and *P* values were generated using voom in the R package limma (67). A Benjamini-Hochberg correction was used to account for multiple comparisons and reported as *P*_adj_. *P*_adj_ < 0.05 was deemed significant.

### Study approval.

All experimental protocols were approved by the UCSD IACUC, and the animals were handled in accordance with the Institutional Guidelines on the Use of Live Animals for Research and ARRIVE (Animal Research: Reporting of In Vivo Experiments) guidelines.

### Data availability.

Raw sequencing data (16S and WGS) were submitted to Sequence Read Archive and are available under the accession no. PRJNA1012006. Data values used to generate each figure are provided in the Supporting Data Values file. The code used for analysis will be provided upon request.

## Author contributions

VSE wrote the manuscript. VSE, JCG, and SKN edited the manuscript. VSE and JCG conducted experiments and acquired data. VSE and JCG analyzed data. SKN provided reagents and resources. SKN conceived of the project. SKN designed various aspects of the research studies. All authors reviewed the manuscript.

## Supplementary Material

Supplemental table 1

Supporting data values

## Figures and Tables

**Table 1:**
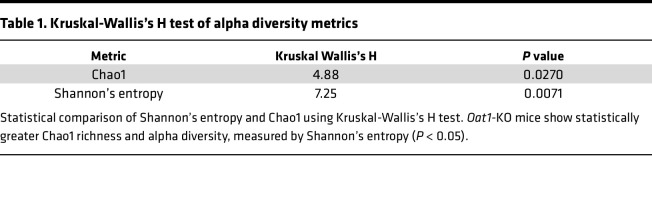
Kruskal-Wallis’s H test of alpha diversity metrics

**Table 2:**
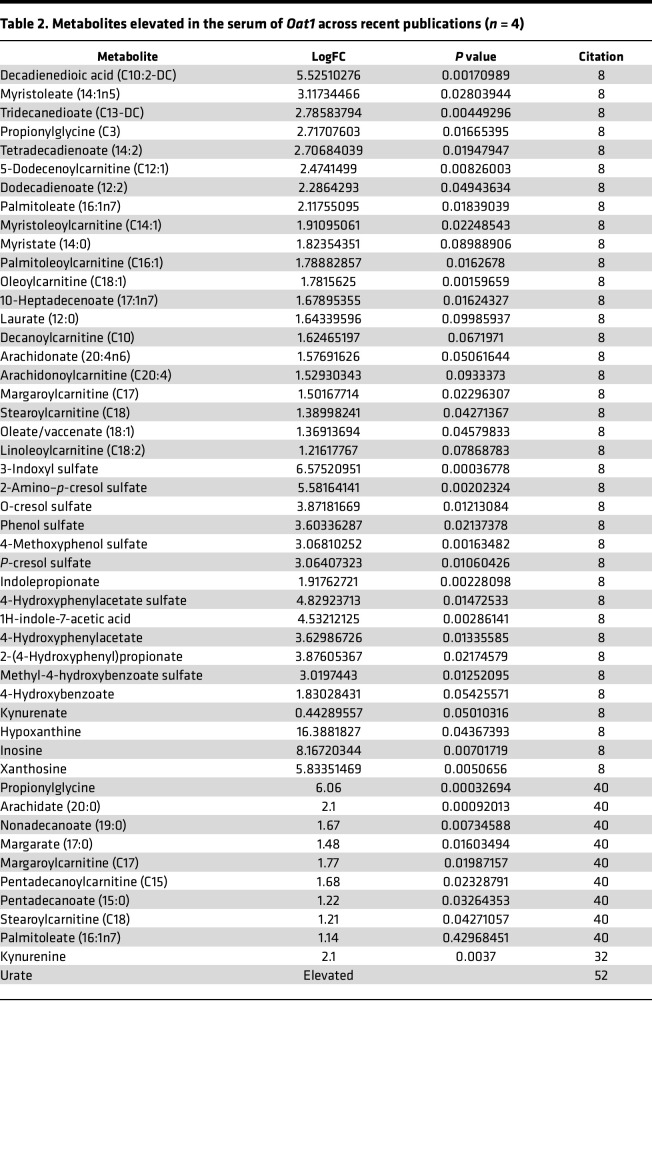
Metabolites elevated in the serum of *Oat1* across recent publications (*n* = 4)

## Version 1 11/08/2023

Electronic publication
